# The extracellular matrix protein EMILIN-1 impacts on the microenvironment by hampering gastric cancer development and progression

**DOI:** 10.1007/s10120-024-01528-z

**Published:** 2024-06-28

**Authors:** Alessandra Capuano, Maddalena Vescovo, Simone Canesi, Eliana Pivetta, Roberto Doliana, Maria Grazia Nadin, Masami Yamamoto, Tetsuya Tsukamoto, Sachiyo Nomura, Emanuela Pilozzi, Antonio Palumbo, Vincenzo Canzonieri, Renato Cannizzaro, Eugenio Scanziani, Gustavo Baldassarre, Maurizio Mongiat, Paola Spessotto

**Affiliations:** 1 Molecular Oncology Unit, Centro di Riferimento Oncologico Aviano, (CRO) IRCCS, Via Franco Gallini 2, 33081 Aviano, PN Italy; 2 Dipartimento di Medicina Veterinaria e Scienze Animali (DIVAS), Università Degli Studi di Milano, Milan, Italy; 3 Oncological Gastroenterology Unit, Centro di Riferimento Oncologico Aviano, (CRO) IRCCS, Aviano, Italy; 4 Laboratory of Physiological Pathology, Nippon Veterinary and Life Science University, Tokyo, Japan; 5 Department of Pathology, Graduate School of Medicine, Fujita Health University, Toyoake, Japan; 6 Department of Clinical Pharmaceutical Sciences, School of Pharmacy and Pharmaceutical Sciences, Hoshi University, Tokyo, Japan; 7 Department of Clinical and Molecular Medicine, Sapienza University of Rome, Azienda Ospedaliero-Universitaria Sant’Andrea, Rome, Italy; 8 Pathology Unit, Centro di Riferimento Oncologico Aviano, (CRO) IRCCS, Aviano, Italy; 9 Department of Medical, Surgical and Health Sciences, University of Trieste, Trieste, Italy; 10 Present Address: Clinical Pathology Unit, Ospedale Santa Maria Degli Angeli, Pordenone, Italy

**Keywords:** Mouse models, Extracellular matrix, Lymphatic vessels, Tumor microenvironment, Gastrointestinal intraepithelial neoplasia

## Abstract

**Background:**

The contribution of the tumor microenvironment and extracellular matrix to the aggressive biology of Gastric Cancer (GC) has been recently characterized; however, the role of EMILIN-1 in this context is unknown. EMILIN-1 is an essential structural element for the maintenance of lymphatic vessel (LV) integrity and displays anti-proliferative properties as demonstrated in skin and colon cancer. Given the key role of LVs in GC progression, the aim of this study was to investigate the role of EMILIN-1 in GC mouse models.

**Methods:**

We used the syngeneic YTN16 cells which were injected subcutaneously and intraperitoneally in genetically modified EMILIN-1 mice. In alternative, carcinogenesis was induced using *N*-Methyl-*N*-nitrosourea (MNU). Mouse-derived samples and human biopsies were analyzed by IHC and IF to the possible correlation between EMILIN-1 expression and LV pattern.

**Results:**

Transgenic mice developed tumors earlier compared to WT animals. 20 days post-injection tumors developed in EMILIN-1 mutant mice were larger and displayed a significant increase of lymphangiogenesis. Treatment of transgenic mice with MNU associated with an increased number of tumors, exacerbated aggressive lesions and higher levels of LV abnormalities. A significant correlation between the levels of EMILIN-1 and podoplanin was detected also in human samples, confirming the results obtained with the pre-clinical models.

**Conclusions:**

This study demonstrates for the first time that loss of EMILIN-1 in GC leads to lymphatic dysfunction and proliferative advantages that sustain tumorigenesis, and assess the use of our animal model as a valuable tool to verify the fate of GC upon loss of EMILIN-1.

## Introduction

Gastric cancer (GC) is a widespread malignant carcinoma worldwide [1]. Despite the improvement of surgical techniques and adjuvant therapy, GC is one of the most common causes of cancer-related deaths, likely due to the late diagnosis but also for the high incidence of recurrence. GC progression is characterized by striking changes in the lymphatic vasculature which promote GC metastasization. In fact, lymph node metastasis is the most common feature during GC spread, and represents the most important prognostic factor determining the clinical outcome [2]. The intricate interplay between processes, such as inflammation, lymphangiogenesis, extracellular matrix (ECM) remodeling, and chemotactic signaling within the GC microenvironment, can deeply affect lymphatic metastasis although the mechanisms are not fully understood. However, it is known that these complex interactions create a permissive microenvironment enabling cancer cells to invade lymphatic vessels (LVs) and disseminate to regional lymph nodes, ultimately impacting on disease progression and patient outcome [3, 4]. Moreover, impaired lymphangiogenesis, which normally facilitates the resolution of inflammation and promotes the clearance of inflammatory cells, can lead to a variety of consequences, including persistent inflammation which eventually exacerbates GC cell malignancy [5]. Compelling evidences indicate that GC microenvironmental components, such as inflammatory cells, altered LVs, and ECM constituents, actively contribute to the aggressiveness of this malignancy [6, 7]. A functional link between ECM protein EMILIN-1, tumor growth and lymphangiogenesis has been extensively demonstrated in different tumor models [8–10]. But a possible role in the contest of GC has not been determined yet. EMILIN-1 is a trimeric multidomain protein known to affect TGF-β maturation via the N-terminal EMI domain [11]. It exhibits anti-proliferative effects through the interaction of the E954 residue of the gC1q domain with α4β1 and α9β1 integrins [12–14], and represents an essential structural component for LV maintenance and function [8, 15, 16]. Interestingly, the finding that α4 integrin subunit is transcriptionally repressed in GC cell lines, but not in the normal gastric epithelium [17], suggests a possible mechanism exerted by integrin/EMILIN-1 recognition in the repression of cell proliferation in this malignancy.

Indeed, in other tumor models, we found that the absence of EMILIN-1 accelerates tumor development and increases the number and size of colon tumors, while its expression halts cell proliferation and counteracts lymphatic dysfunction in the AOM/DSS model of inflammatory colon carcinogenesis [9].

In this study, we tested whether EMILIN-1 could also play an oncosuppressive role in the gastric microenvironment, where inflammation, lymphangiogenesis, and alterations in ECM components represent a relevant pathogenic mechanism.

## Materials and methods

### Animals and in vivo procedures

C57BL/6 J mice were purchased from Charles River Laboratories. The *Emilin1*^*−/−*^ (KO) [15, 16, 18] and *Emilin1*-E955A (E955A-KI) (formerly referred as E933A-TG [8, 9, 19, 20]) corresponding to the E954 in the human hortolog, mouse models were generated and maintained at the CRO-IRCCS mouse facility. All animal procedures and their care were performed according to the institutional guidelines in compliance with national laws and with the authorizations by the Italian Ministry of Health to Dr. Spessotto (n°632/2020) and Dr. Capuano (n°348/2023 PR).

#### Injection of the syngeneic YTN16 cell line [21]

5 × 10^6^ YTN16 cells were subcutaneously injected into each flank of 8-week-old WT, KO and E955A-KI mice. Tumor growth was monitored every other day and animals were sacrificed after 20 days. To evaluate the dissemination ability, 10 × 10^6^ YTN16 cells were intraperitoneally injected; mice were sacrificed 20 days later.

#### Chemically induced gastric carcinogenesis model

Proliferative lesions of the mouse stomach were induced according to a previously reported procedure [22]. In brief, 6-week-old mice were given drinking water ad libitum containing 240 ppm MNU (*N*-Methyl-*N*-nitrosourea) (Selleckchem) in light-shielded bottles. MNU was administered alternately with saline (NaCl) for 10 weeks, followed by a 20-week-period in which the animals received normal water. Mice were sacrificed after 22 weeks. MNU was dissolved in distilled water and freshly prepared 3 times per week.

### Histopathology, immunohistochemistry, and immunofluorescence analyses of mouse tissues

Samples of various organs and tissues were fixed in 10% neutral buffered formalin for 48 h, transferred in 70% ethanol and processed for paraffin embedding; 5 µm-thick sections were obtained and stained with hematoxylin and eosin (H&E). Samples were examined with a light microscope for the detection and quantification of proliferative lesions and other histological features. Gastric proliferative lesions were classified according to Nolte et al. [23] with some modifications as follows: 1. Proliferative lesions: hyperplasia (increased number of normal epithelial cells); 2. Pre-neoplastic lesions: hyperplasia with atypia/dysplasia (increased number of cells characterized by atypia and pleomorphism/abnormal structure of gastric glands); 3. Neoplastic lesions (epithelial): (a) adenoma; (b) gastrointestinal intraepithelial neoplasia (GIN, focal intraepithelial atypical proliferations resembling morphologically an adenocarcinoma).

To assess tumor spread after intraperitoneal injection of YTN16 cells, the total score was calculated as the sum of tumor deposits found in six different regions (1. spleen, liver kidneys; 2. lung, heart, thymus; 3. stomach, duodenum, pancreas; 4. cecum, ileum, colon, mesentery; 5. uterus, ovaries; 6. diaphragm, abdominal wall) and classified depending on the size of the lesion. An histopathological score was assigned for each metastatic lesion according to the following criteria: small (< 20 neoplastic cells): 1 point; medium > 20 neoplastic cells but maximum 2 mm in diameter): 2 points; large (> 2 mm in diameter): 4 points.

The stomach from mice submitted to the MNU protocol was stained immunohistochemically with the following primary antibodies: Ki-67 (Thermo Scientific), and γH2AX (Cell Signaling).

For IF analyses of gastric paraffin-embedded samples, the anti-podoplanin (Abcam) and anti-EMILIN-1 (Abcam) antibodies were employed. Mouse subcutaneous YTN16-derived tumors were OCT embedded and analyzed for blood and lymphatic vessels using anti-CD31 (BD) and anti-Lyve-1 (Abcam). Appropriate Alexa Fluor® 488/568-conjugated secondary antibodies (Life Technologies) were employed; TO-PRO™ (Life Technologies) was used to visualize nuclei. Images were acquired with a confocal scanner system (TCS SP8 FSU AOBS, Leica Microsystems), equipped with a Leica DMi8 inverted microscope (Leica Microsystems). At least 5 fields (20X, original magnification) were acquired and quantification of the fluorescence positive structures was assessed by means of the Volocity software (PerkinElmer Inc., Waltham, MA, USA).

### Patients

Gastric biopsies were obtained during endoscopy from 20 patients from the Oncologic Gastroenterology Unit of CRO-IRCCS Aviano (Italy) who had given written informed consent. This study was approved by the Institutional Board of CRO-IRCCS Aviano (IRB n°CRO-2014-03). Experienced gastroenterologists and pathologists classified the lesions according to endoscopic and histopathologic diagnostic criteria. Adjacent healthy tissues were used as a control. Patients were of both sexes and similar age. Intestinal and diffuse histotypes were equally represented. Publicly available GSE66229 and GSE26253 data set were queried for the expression of EMILIN-1, podoplanin, MMP9 and MMP14. The following probe sets were used: *EMILIN1*, 204163_at; *PDPN*, 208233_at; *MMP9*, 203936_s_at; and *MMP14*, 202827_s_at. The TNMplot tool (https://tnmplot.com/analysis) [24] was also interrogated to evaluate the expression of EMILIN-1 in a spectrum of tumor types.

### Immunofluorescence analysis of human gastric biopsies

For IF analyses on human bioptic samples, serial cryostat sections (7 μm) were collected. The sections were equilibrated at room temperature, hydrated with PBS for 5 min, and fixed with PBS 4% PFA for 15 min. Then, they were permeabilized for 5 min and saturated with the blocking buffer (PBS, 1% BSA, and 2% FCS) for 30 min. The primary antibodies were then incubated at room temperature for 1 h. The slices were stained using the mouse monoclonal anti-human podoplanin (D240 clone, Abcam), the rabbit polyclonal anti-human EMILIN-1 As-556 (produced in our laboratories, as previously described [25–27]), and the rabbit polyclonal antibody against elastase (Abcam) to visualize neutrophils. After three 5-min washes in PBS, the appropriate Alexa Fluor^®^ 488/568-conjugated secondary antibodies were employed and TO-PRO™ was used to visualize nuclei. Images were acquired with a confocal scanner system (TCS SP8 FSU AOBS).

### Evaluation of the inflammatory infiltrate

The presence of neutrophils and lymphocytes, easily recognized by the morphology of their nuclei, was evaluated in H&E-stained paraffin-embedded sections from both MNU mouse model and tumor bioptic human samples. To detect macrophages, mouse and human sections were stained immunohistochemically with anti Iba1 (Dako) and anti CD68 (Dako) antibodies, respectively. In both mouse and human samples, the presence of inflammatory cells was scored as follows: absent (no infiltrate); rare (slight); some (mild); numerous (severe).

### Statistical analysis

Graphpad Prism (version 7.02) was used for statistical analyses. The t-test was used when the values were normally distributed. *P*-values < 0.05 were considered significant. All measurements are shown as mean ± SD.

## Results

### An altered EMILIN-1 expression promotes GC growth and lymphatic vessel anomalies

The finding that the ECM molecule EMILIN-1 exerts a homeostatic effect in the skin and colon [9, 10, 13] prompted us to assess whether its normal expression and functionality are also necessary for the control of cell proliferation in the gastric environment.

**Fig. 1:**
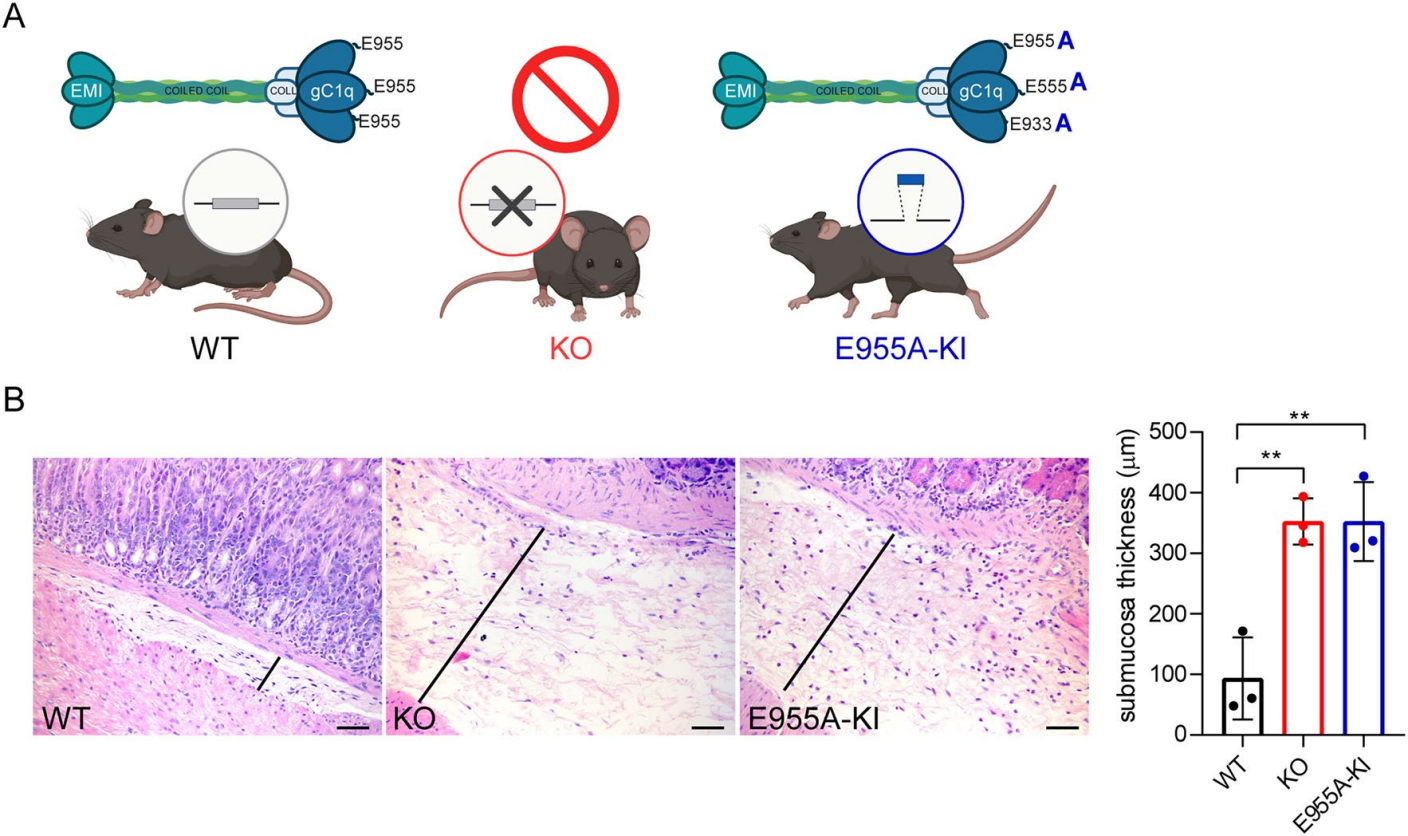
EMILIN-1 transgenic mice display gastric mucosa thickening. **A** Schematic representation of the EMILIN-1 in vivo models: the wild-type C57BL/6 J mouse (WT), the EMILIN-1 knockout mouse (KO) and the knock-in mouse carrying the E955A mutation in the gC1q domain (E955A-KI) (created with BioRender.com). **B** H&E staining of normal gastric mucosa from 10-month-old mice. On the right, graph reporting the measurements of submucosa thickness. Note the presence of edema in KO and E955A-KI submucosa. The results represent the mean ± SD of 3 mice/genotype. The black lines indicate the range of measurement evaluated (at least 5 measurements/mouse). *P*-values were calculated using the two-tailed Student’s t-test. **P < 0.01. Scale bar = 50 µm

**Fig. 2:**
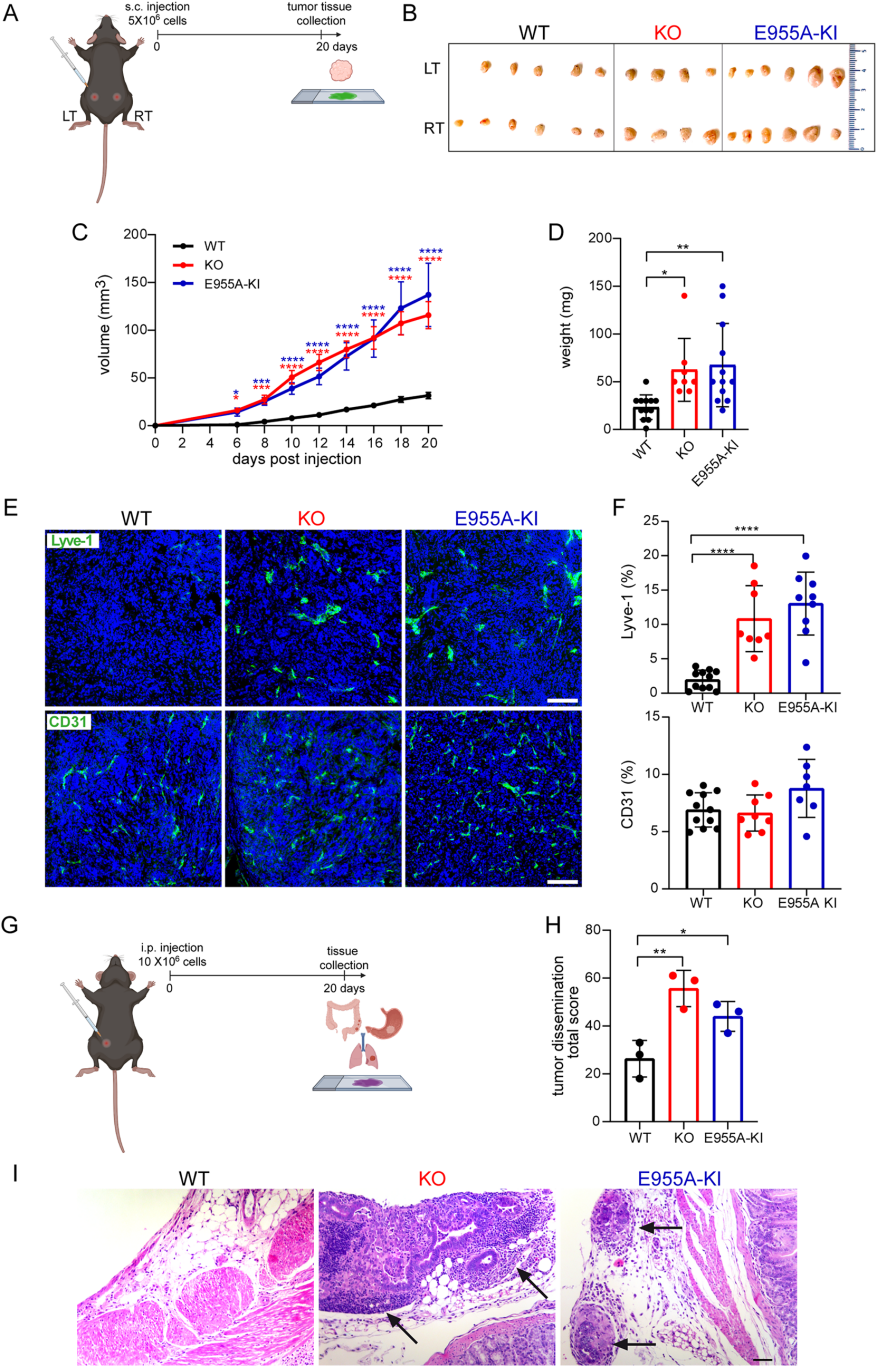
Loss of EMILIN-1 function promotes GC growth and tumor spread. **A** Schematic representation of the experimental procedure for the subcutaneous (s.c.) injection. **B** Tumor masses developed 20 days after s.c. injection of YTN16 murine GC cells in both flanks (LT, left and RT, right) of WT (n = 6), KO (n = 4) and E955A-KI (n = 6) mice. Volume curves (**C**) and endpoint weight (**D**), expressed as mean ± SD, show accelerated and increased tumor growth in transgenic mice. **E** Representative immunofluorescence images of cryostat sections of tumors developed in WT, KO and E955A-KI mice. KO and E955A-KI have higher lymphatic vessel density compared to WT tumors, as shown by Lyve-1 staining. The density of blood vessels is similar in the three genotypes, as shown by CD31 staining. The corresponding graphs (**F**) report the Lyve-1 and CD31 positive volume calculated with the Volocity software in the entire analyzed sections (20x). For each sample, at least 5 fields were analyzed and the mean value was reported. Results represent the mean ± SD of 8–12 tumors/genotype. **G** Schematic representation of the experimental procedure for intraperitoneal (i.p.) injection. **H** Quantification of the total score used to evaluate tumor spread (see “Materials and Methods”) and shown in the graph as mean ± SD of n = 3 mice per group. **I** Representative images of the gastric serosal wall showing the spread of YTN16 cells 20 days after i.p. injection in WT, KO, and E955A- KI mice. Black arrows indicate tumor masses. Schemes in (**A**) and (**G**) have been created with BioRender.com. *P*-values were calculated using a two-tailed Student’s t-test. *P < 0.05, **P < 0.01, ****P < 0.001. Scale bar = 50 µm

To this end, we performed in vivo experiments with various mouse models developed in our laboratory. These models include the C57BL/6 J wild-type mouse (WT), the *Emilin-1* knockout mouse (KO), and the knock-in mouse carrying a mutant form of EMILIN-1 (E955A-KI) in the gC1q domain, which prevents the binding to the α4- and α9-integrins (Fig. 1A) and allows to distinguish between integrin-dependent and -independent effects. We found that the aged transgenic animals showed thickness of the gastric submucosa associated with edematous tissue suggesting possible lymphatic stasis (Fig. 1B). In addition, a more pronounced inflammatory infiltrate was observed in the submucosa of KO and E955A-KI mice (Fig. 1B), suggesting that the microenvironment in these animals may represent a favorable niche for tumor development. To assess in detail the tumor development in the different genotypes, we performed in vivo experiments using the GC syngeneic YTN16 cell line [21]. Following subcutaneous injection of YTN16 cells, tumors developed earlier in KO and E955A-KI mice and were larger compared to those grown in WT mice (Fig. 2A–D). In fact, in KO and E955A-KI animals, palpable tumors appeared on both backs already at 6 days following the inoculation of cancer cells (Fig. 2C). Tumors from the YTN16 subcutaneous model were stained with the anti-Lyve-1 and anti-CD31 antibodies to identify lymphatic and blood vessels. While no differences were observed using the anti-CD31 antibody, a statistically significant increase of intratumoral lymphangiogenesis was detected in KO and E955A-KI animals (Fig. 2E and F).

The tumor-suppressive function of EMILIN-1 was corroborated also through the intraperitoneal injection of YTN16 cells in transgenic mice. In fact, twenty days following injection, we detected larger and more numerous tumor masses in KO and E955A-KI mice respect to WT animals (Figs. 2G–I). To quantitatively assess the extent of metastasis, an endpoint score was determined based on the extent of tumor spread to different organs. The total score was significantly higher in transgenic mice, especially in the peritoneum, where the presence of nodules was more evident than in the WT animals (Fig. 2H).

**Fig. 3:**
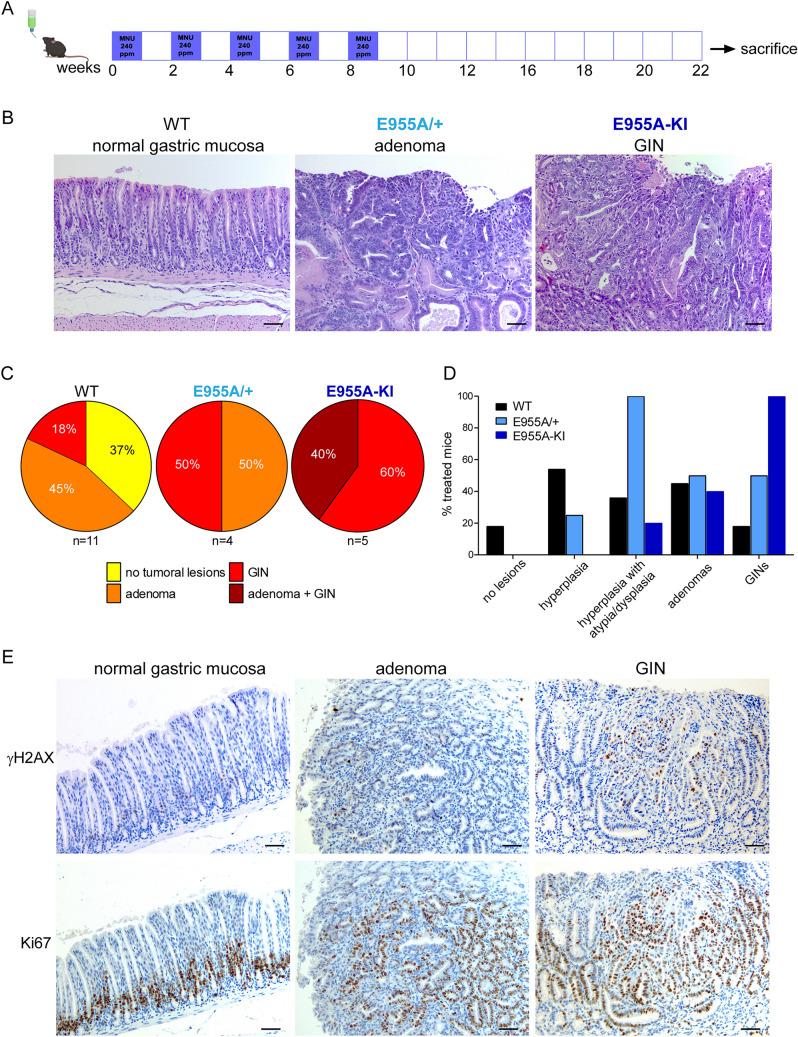
Gastric Carcinogenesis induced by MNU is more pronounced in EMILIN-1 transgenic mice. **A** Scheme of administration regimen of *N*-Methyl-*N*-nitrosourea (MNU) to the animals (created with BioRender.com). Representative H&E images of the most common lesions (adenomas and GINs) in the stomach in the three genotypes (**B**) and the relative quantifications (**C**), expressed as a percentage of the total treated mice, as indicated. **D** Graphical representation of the distribution of hyperplasia, atypia, dysplasia and lesions in all treated mice. **E** Representative images of paraffin-embedded serial sections of normal gastric mucosa, adenoma and GIN, immunostained with an anti-γH2AX antibody as a marker for DNA double-strand breaks, and with an anti-Ki-67 antibody as a marker for cell proliferation. Scale bar = 50 µm

To effectively study tumorigenesis and early development of GC, we chemically induced carcinogenesis using *N*-Methyl-*N*-nitrosourea (MNU). Although this model is informative, it requires a long time frame and has low penetration [22]. We chose hence to employ only the E955A-KI transgenic mice since they allow to ascertain if the possible effects depend on EMILIN-1/integrin interaction, which is lost in this model, or TGF-β levels, which are allowed via the intact N-terminal region [8]. Furthermore, for this experimental setup to gain insights into the dominance of the modified allele and its role in tumor development, we also employed heterozygous KI animals (E955A/ +). Mice were administered MNU ad libitum for five weeks, alternating with saline every other week, for a total of ten weeks. At the end of the treatments, the animals were sacrificed and the stomach thoroughly analyzed (Fig. 3A). Treatment with MNU induced the formation of gastrointestinal intraepithelial neoplasia (GIN) and adenomas (Fig. 3B). The frequency of lesion development differed between the genetic backgrounds under study: 37% of WT mice showed no lesions, whereas 45% had adenomas and 18% had GIN; in contrast, all E955A/ + and E955A-KI mice developed at least one type of lesion: 50% of heterozygous mice showed adenomas and the other 50% GIN; 60% of E955A-KI mice showed GIN and the remaining 40% developed both GIN and adenomas (Fig. 3C). Hyperplastic tissue characterized by atypia and/or dysplasia was well represented in all genotypes (Fig. 3D). On the contrary, WT and transgenic mice displayed a marked difference in the number of GIN, which was higher in E955A-KI mice. Comparing the histological features between the lesions, GINs showed more aggressive traits compared to adenomas, being characterized by a higher level of cellular atypia, a higher number of γH2AX-positive cells, suggestive of increased levels of DNA damage, and a markedly increased distribution of Ki-67 positivity (Fig. 3E).

**Fig. 4:**
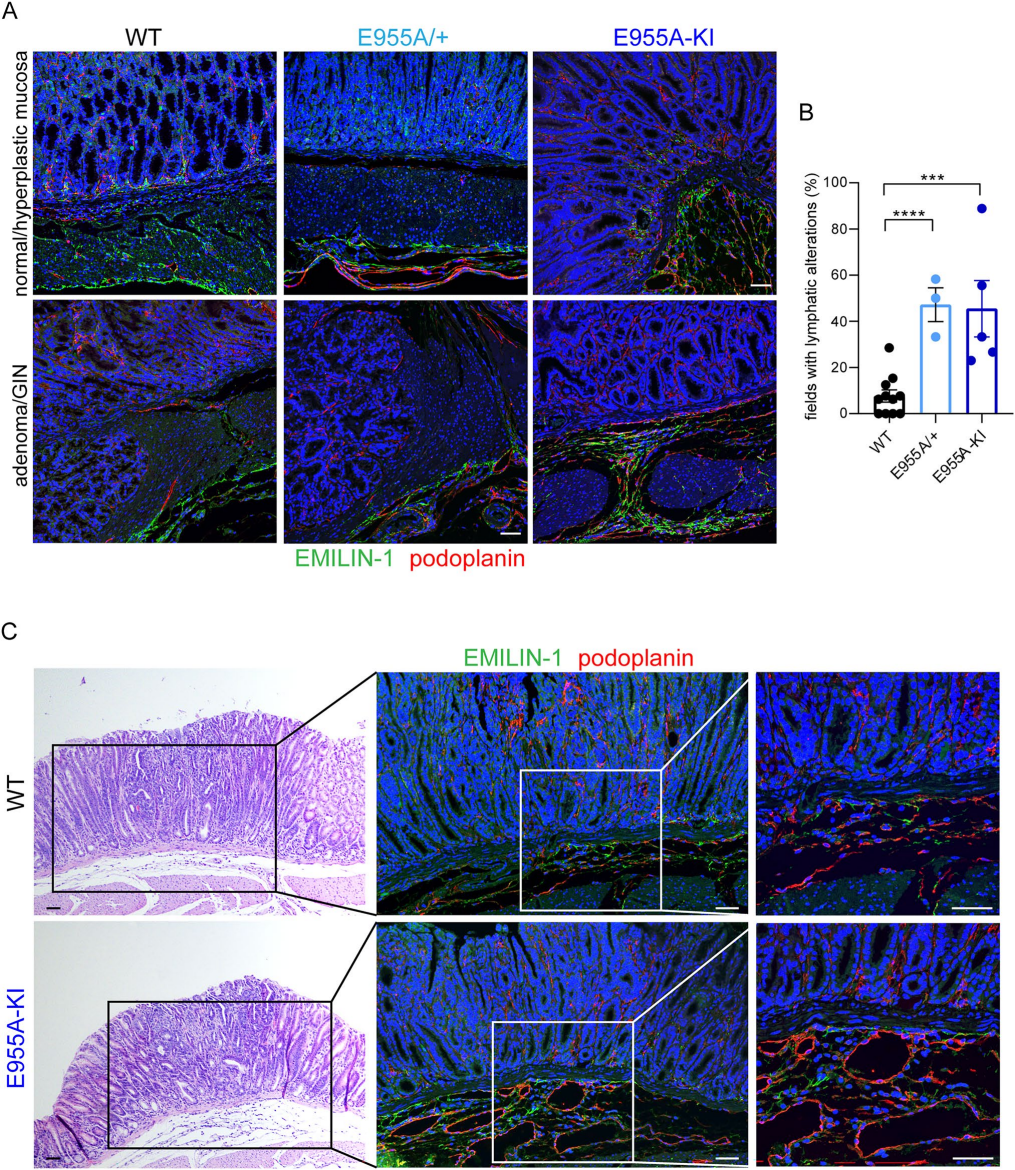
The EMILIN-1 transgenic mice display extensive LV aberrations. **A** Representative images of normal and tumor gastric mucosa and submucosa immunostained with anti-EMILIN-1 (green) and the anti-podoplanin (red) antibodies in MNU-treated mice. **B** Graph showing the frequency of lymphatic changes (assessed as the presence and extent of LVs with dilated lumen in the submucosa) reported as mean ± SD of 11 WT, 3 E955A/ + and 5 E955A-KI mice, as indicated by the number of dots in each histogram. **C** Analysis of LVs (red) in the submucosa of GIN lesions from WT and E955A-KI mice. Paraffin-embedded serial sections were stained with H&E to identify the lesion and immunostained for EMILIN-1 (green) and podoplanin (red). The outlined areas correspond to the region analyzed by immunofluorescence. ***P < 0.005; ****P < 0.001. Scale bar = 50 µm

Importantly, upon treatment, the immunofluorescence analyses showed more extensive morphological changes in the LVs in the transgenic mice compared to those observed in WT animals (Fig. 4A). The presence and extent of LVs with a dilated lumen in the submucosal layer were the criteria used to evaluate lymphatic alterations in all recorded fields of the mouse stomach. The percentage of fields in which this type of morphology was detected in each mouse was significantly higher in both E955A/ + and E955A-KI transgenic animals than in WT mice (Fig. 4B). The lymphatic changes in heterozygous mice compared to E955A-KI mice were negligible, likely due to the inclusion of mutant monomers in the EMILIN-1 trimers, which is known to abrogate the function of the molecule [14], thus resulting in a dominant negative effect.

To verify whether the LV changes were primarily due to impaired EMILIN-1 expression or also due to the higher frequency of lesions in transgenic mice compared to WT mice, we thoroughly examined the morphology of LVs in the submucosal region of the same type of lesions in both genotypes. As shown in Fig. 4C, GINs developed in E955A-KI animals were characterized by LVs with larger lumens in contrast to WT mice.

### EMILIN-1 is downregulated in human GC specimens

**Fig. 5:**
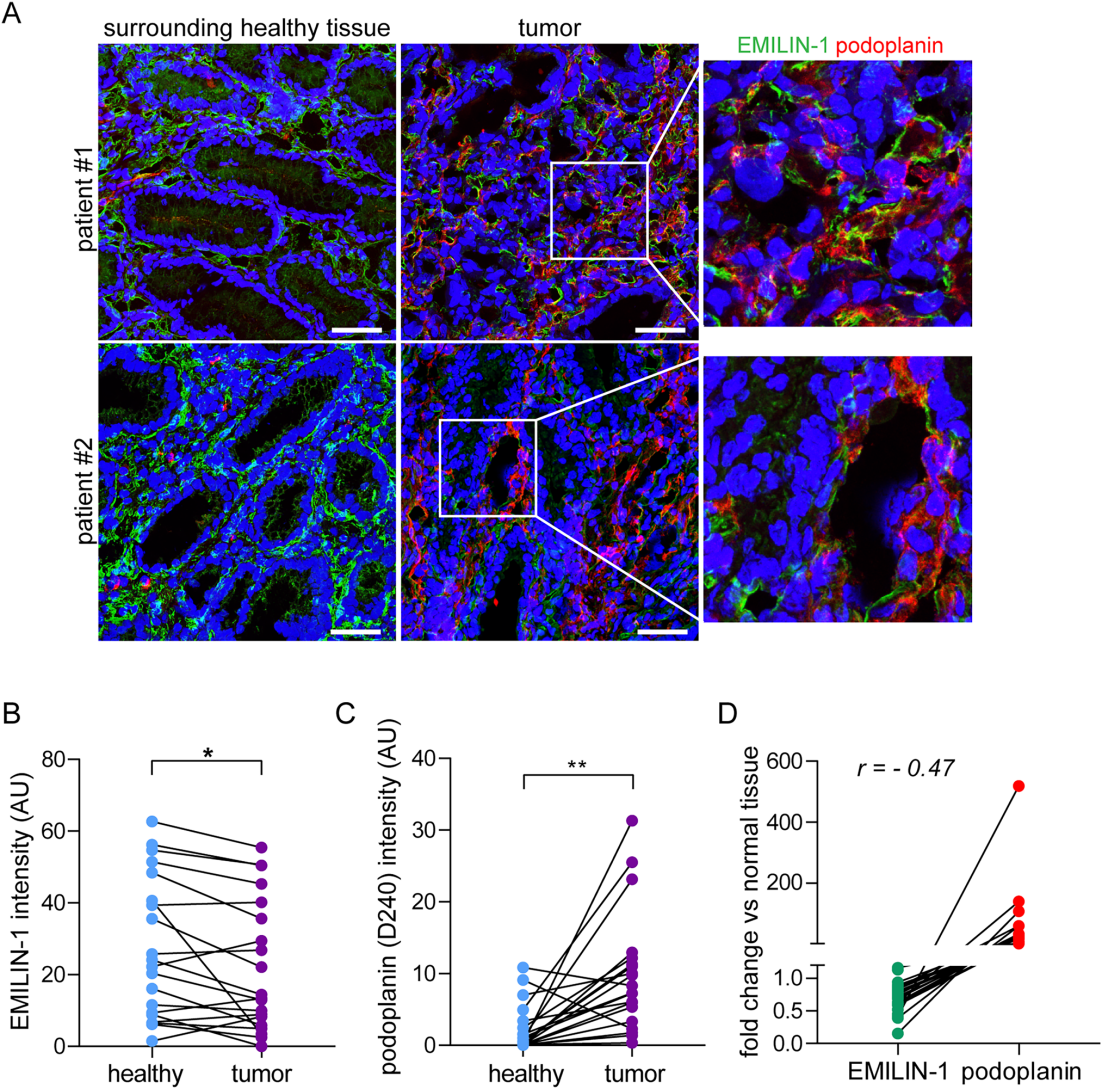
EMILIN-1 expression is downregulated in human GC samples. **A** Representative images of cryostat sections of a tumor and the corresponding adjacent healthy human gastric tissue from two different patients immunostained for EMILIN-1 (green) and podoplanin (red). The enlarged fields on the right correspond to the white outlined areas in the tumor samples and show details of the LVs. Graphs quantifying EMILIN-1 (**B**) and podoplanin (**C**) positive volumes in the entire analyzed sections (20x) of the paired samples. At least 5 fields were analyzed for each sample. 20 GC patients were analyzed and the mean value was reported for each. **D** The graph shows the inverse correlation between the expression of EMILIN-1 and podoplanin in the patient cohort. The fold change was determined by comparing the intensities of the tumor and normal counterpart in paired samples. Pearson’s correlation coefficient was calculated between the two variables. Scale bar = 50 µm. *P*-values were calculated using a two-tailed paired Student’s t-test. *P < 0.05, **P < 0.01

To verify the role of EMILIN-1 and the effects of its loss in lymphatic dysfunction in human GC, we examined the EMILIN-1 levels in biopsy samples from a small cohort of patients. We found that EMILIN-1 was diffusely deposited in the normal human gastric mucosa, whereas its deposition was significantly decreased in malignant samples (Fig. 5A and B). Interestingly, the decreased EMILIN-1 levels in tumor tissue associated with the presence of morphologically aberrant podoplanin-positive LVs (Fig. 5A–D).

**Fig. 6:**
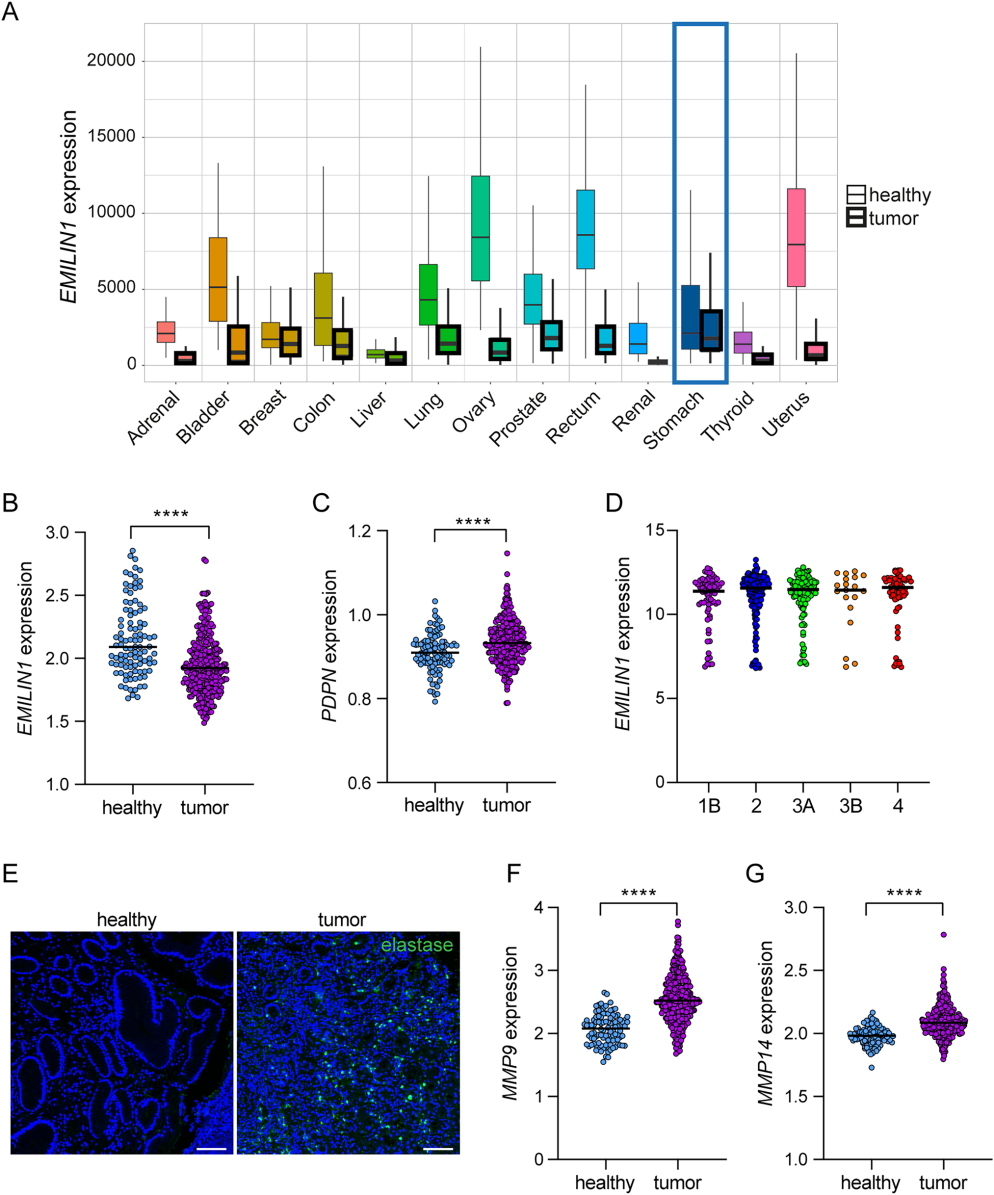
EMILIN-1 expression is downregulated in tumors and inversely associates with podoplanin, elastase, MMP9 and MMP14. **A** Graph showing *EMILIN1* expression in different tumors and the corresponding normal tissue as assessed with the TNMplot tool. The tumors with statistically different values are indicated in the graph. The graph represents the gene expression analysis of *EMILIN1* (**B**) and podoplanin (*PDPN)* (**C**) evaluated with the GEO dataset GSE66229 (healthy, n = 100; tumor, n = 300). **D** Distribution of *EMILIN1* expression at different GC stages (GSE26253; 1B, n = 68; 2, n = 167; 3A, n = 111; 3B, n = 19; 4, n = 67). **E** Representative images of sections of healthy gastric and tumor tissue from our patient cohort, immunostained for elastase (green). Graphical representation of *MMP9* (**F**) and *MMP14* (**G**) expression using the GEO dataset GSE66229. *P-*values were calculated using a two-tailed Student’s t-test. ****P < 0.0001. Scale bar = 50 µm

To gain further insight in this putative relationship, we performed in silico analyses mining data from different dataset. Interestingly, the use of the online TNMplot tool revealed an overall and significantly decreased EMILIN-1 expression in several tumors, including GC (Fig. 6A). A substantial decrease of EMILIN-1 expression was observed in carcinomas of the bladder, lung, ovary, rectum and uterus respect to the normal tissues (Fig. 6A), suggesting that the EMILIN-1 tumor-suppressive function may be lost in a broad spectrum of cancers. Similar results were obtained interrogating the GSE datasets (Fig. 6B). Interestingly, the decrease of EMILIN-1 expression associated with an increase of podoplanin expression (Fig. 6C). Remarkably, analysis of another dataset that included GC at different stages showed no variations in EMILIN-1 expression, suggesting that its levels could be particularly critical in the early phases of the disease but irrelevant during tumor progression (Fig. 6D). To test whether the reduced EMILIN-1 protein levels (Fig. 5) were not only a consequence of transcriptional regulation but also the result of a degradation process, we examined the presence of neutrophil elastase, a protease known to process EMILIN-1 [27, 28]. Indeed, we observed positive and abundant staining in the GC mucosal microenvironment (Fig. 6E). In addition, the expression of MMP9 and MMP14, two other enzymes known to degrade EMILIN-1 [28], was significantly increased in GC lesions (Fig. 6F and G).

### The inflammatory infiltrate in tumor lesions is associated with LV changes

**Fig. 7:**
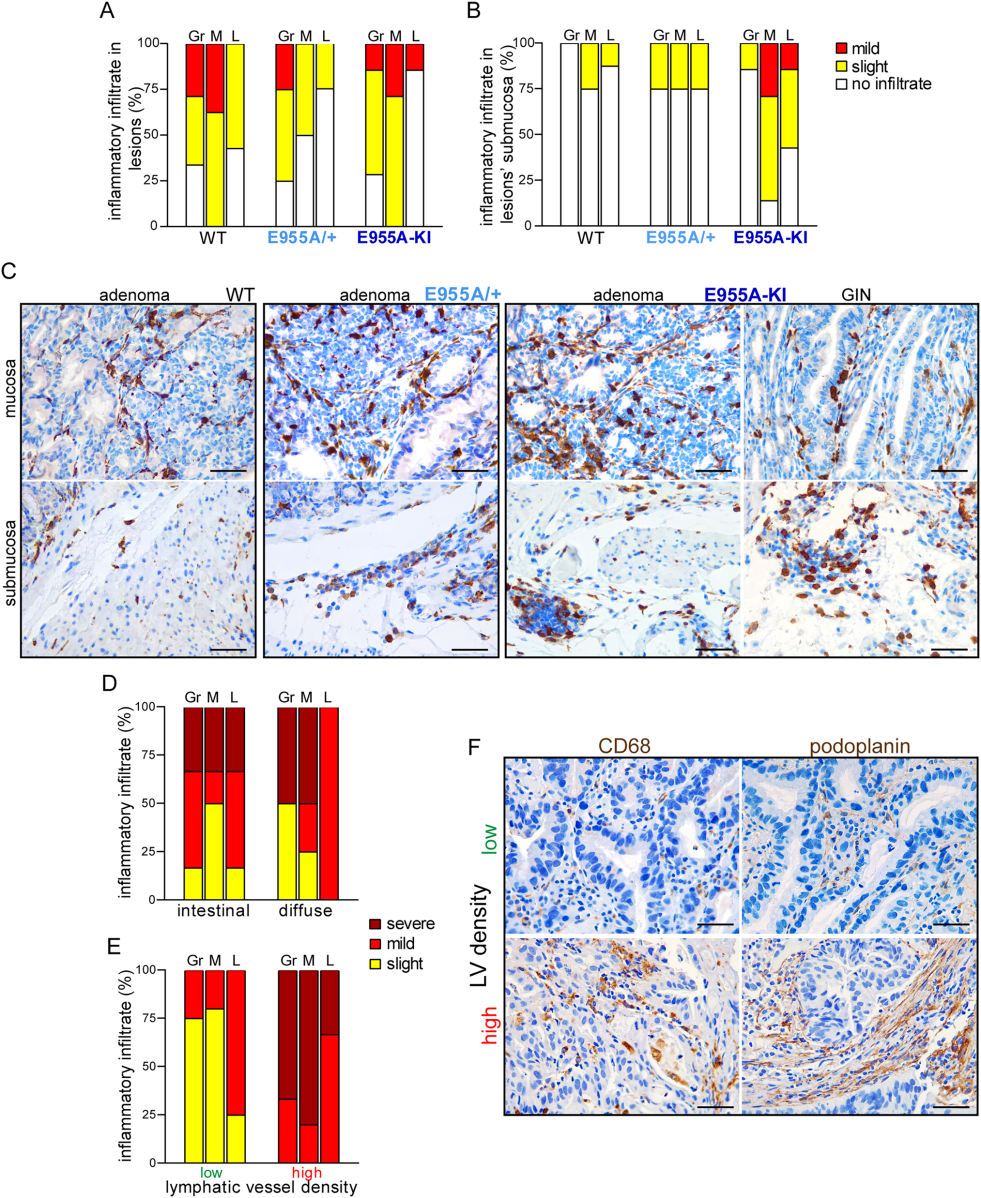
The inflammatory infiltrate in tumor lesions is associated with LV anomalies and density. Graphical distribution of the inflammatory infiltrate in tumor lesions (**A**) and in the corresponding submucosa (**B**) in MNU-treated animals. Gr, granulocytes/neutrophils; M, macrophages; L, lymphocytes. **C** Representative images of Iba1 staining of tumor mucosa (top) and submucosa (bottom) in WT, E955A/ + and E955-KI mice. Qualitative analysis of inflammatory infiltrate in the bioptic samples of GC patients grouped by histotype (**D**) or LV density (E). LV density was scored by podoplanin-positive staining in 20 × fields as follows: low (< 10 LVs/field), high (≥ 10 LVs/field). F) Representative images of IHC staining for the detection of macrophages (CD68) (left) and LVs (podoplanin) (right) in human tumor samples. One patient is shown for each of the “low” and “high” LV density groups. Scale bar = 50 µm

The analyses of inflammatory infiltrates also showed a correlation between lymphatic dysfunction and tumor growth due to decreased EMILIN-1 levels. In the animal lesions (adenomas and GINs), there were no significant differences in the amount of infiltrate and its composition (with the prevalence of granulocytes and macrophages) among the three mouse genotypes (Fig. 7A). Interestingly, the submucosa of the lesions was more infiltrated in the transgenic mice (Fig. 7B and C), and aberrant lymphatic vessels were easily recognizable there in association with edema (Fig. 4). Regardless of histotype, a stronger infiltrate was observed in human samples, which have a high density of LVs (Fig. 7D–F).

## Discussion

Successful treatment of GC still represents a challenge in cancer therapy and this is in part due to the poor understanding of the intricate players within the tumor microenvironment [29]. ECM remodeling plays a central role in cancer invasion and metastasis, and was also demonstrated influence different steps of GC development [30]. For example, increased expression of tenascin has been shown in pre-malignant and malignant gastric epithelia [31], while collagens have been demonstrated to be generally upregulated at more advanced stages [32–34]. Of note, a subset of collagen genes was reported to be a potential marker to distinguish pre-malignant from malignant lesions [34]. Considering that some ECM components have been shown to function as promising potential prognostic biomarkers, it is essential to identify the putative molecules that may serve to improve the management of GC patients. Several recent studies have focused on specific molecular signatures using proteomic and integrative bioinformatic approaches. The studies have further emphasized the need to better characterize the ECM proteins and their distribution in order to identify new approaches to improve prognosis, response to the therapies and the GC patients’ outcome [35–40]. Unfortunately, a clinical validation is still missing for many candidates. Also the mechanisms of action of these molecules that may be useful for the development of new therapeutic targets are not yet fully understood, possibly due to the complexity of the interactions that these molecules engage and also for the fact that there are few suitable animal models specifically targeting components of the microenvironment or ECM. Most mouse models used to study gastric tumorigenesis involve manipulation of genes leading to the overexpression or deficiency of growth factors, as well as mutations of tumor suppressors and oncogenes [41, 42]. Another active research topic aimed at better understanding the players in GC development concerns the study of the mechanisms regulating LV formation. To identify the major players affecting lymphangiogenesis and contributing to disease progression is essential for the development of targeted therapies that can limit metastasis [4]. In this context, our study provides new pre-clinical and clinical evidence demonstrating a link between loss of EMILIN-1 expression and increased GC growth and abnormal lymphangiogenesis, and it evaluates the use of in vivo animal models as powerful tools for these studies.

Loss of EMILIN-1 has previously been linked to neoplastic progression in skin and colorectal cancer models [9, 10, 43]. Collectively, the findings of the present study suggest that tumor-suppressive function of EMILIN-1 may be abrogated by multiple mechanisms in the context of GC, ultimately contributing to tumor growth and possibly cancer spread via the aberrant lymphatics. Indeed, EMILIN-1, besides protecting the functional integrity of LVs, may act as a sentinel in the gastric microenvironment by exerting an anti-proliferative and oncosuppressive activity. A striking finding of the present study is the association between the presence of aberrant LVs in conjunction with exacerbated gastric tissue malignancy. The pronounced increase of lymphangiogenesis in the tumors from transgenic mice suggests that EMILIN-1 may play a central role in GC progression, LVs being the preferential route for GC cell dissemination [8, 10].

Using genetically modified EMILIN-1 mice, we found that transgenic animals were more prone to develop larger tumors after injection of the syngeneic YTN16 cells, suggesting that the anti-proliferative effect of EMILIN-1 is exerted through the gC1q domain. The significantly increased intratumoral lymphangiogenesis in EMILIN-1 mutant animals further demonstrates the importance of this ECM protein in regulating LV formation and suggests that its loss in GC may profoundly impact on its progression and spread in different organs. The relationship between the structural integrity of EMILIN-1 and functional properties of LVs was further defined when we treated mice with MNU, a potent carcinogen whose efficiency in tumor induction is fairly low according to the literature [22]. Treatment with MNU leads to the formation of well or moderately differentiated adenocarcinomas mainly located in the antrum. In our models, we observed the development of adenomas and GINs, with marked differences between the genetic backgrounds. While WT mice displayed a limited number of lesions, both heterozygous and homozygous E955A mice developed numerous lesions, especially GIN, which, considering the higher levels of DNA damage and Ki-67 positivity, is a more aggressive subtype compared to adenoma. These observations provide important insights into the effects of EMILIN-1 in the early stages of GC development.

The MNU-induced tumors are rich in stromal cells and occasionally invade the submucosa [42, 44]. This model has been used to study the diffuse-type of GC, where the stroma plays an important role [45, 46]; however, we consider that the MNU protocol could also be useful to study the role of ECM and the altered interactions between the epithelial and stromal components of the gastric mucosa occurring during GC formation. The application of the NMU carcinogenesis protocol to the EMILIN-1 transgenic mice was found to be a reliable model to study the role of ECM components, especially in the early stages of carcinogenesis. A very interesting finding obtained in this study is the higher level of LV abnormalities in transgenic mice and their association with more aggressive lesions, suggesting that the pattern of lymphatic vasculature actively contributes to the biology of the neoplastic outbreak [47]. The levels of EMILIN-1 and podoplanin in human samples confirm the goodness of our mouse models.

The loss of EMILIN-1 or its functional impairment could indirectly exacerbate the inflammatory status [9, 19]. In a previous work, we have shown that the absence of EMILIN-1 favors a pro-inflammatory environment with a greater recruitment of macrophages [19]. In this study, we confirm in the gastric tumor microenvironment that the reduced levels of EMILIN-1 or its functional impairment could promote the conspicuous presence of inflammatory cells, probably as a consequence of reduced clearance by the dysfunctional LVs.

The preliminary data on *MMP* expression and the presence of neutrophil elastase in tumor tissue suggest that the decreased EMILIN-1 levels may be due to both transcriptional and enzymatic processes. Further studies should elucidate the mechanisms by which EMILIN-1 is downregulated in GC.

In summary, this study provides new evidence for the emerging role of the ECM in lymphangiogenesis and GC development and opens new insights for the development of new tools to improve the management of GC patients.
